# Galiellalactone Inhibits Stem Cell-Like ALDH-Positive Prostate Cancer Cells

**DOI:** 10.1371/journal.pone.0022118

**Published:** 2011-07-11

**Authors:** Rebecka Hellsten, Martin Johansson, Anna Dahlman, Olov Sterner, Anders Bjartell

**Affiliations:** 1 Division of Urological Cancers, Department of Clinical Sciences, Lund University, Malmö, Sweden; 2 Division of Organic Chemistry, Department of Chemistry, Lund University, Lund, Sweden; University of Bergen, Norway

## Abstract

Galiellalactone is a potent and specific inhibitor of STAT3 signaling which has been shown to possess growth inhibitory effects on prostate cancer cells expressing active STAT3. In this study we aimed to investigate the effect of galiellalactone on prostate cancer stem cell-like cells. We explored the expression of aldehyde dehydrogenase (ALDH) as a marker for cancer stem cell-like cells in different human prostate cancer cell lines and the effects of galiellalactone on ALDH expressing (ALDH+) prostate cancer cells. ALDH+ subpopulations were detected and isolated from the human prostate cancer cell lines DU145 and long-term IL-6 stimulated LNCaP cells using ALDEFLUOR® assay and flow cytometry. In contrast to ALDH− cells, ALDH+ prostate cancer cells showed cancer stem cell-like characteristics such as increased self-renewing and colony forming capacity and tumorigenicity. In addition, ALDH+ cells showed an increased expression of putative prostate cancer stem cell markers (CD44 and integrin α2β1). Furthermore, ALDH+ cells expressed phosphorylated STAT3. Galiellalactone treatment decreased the proportion of ALDH+ prostate cancer cells and induced apoptosis of ALDH+ cells. The gene expression of *ALDH1A1* was downregulated in vivo in galiellalactone treated DU145 xenografts. These findings emphasize that targeting the STAT3 pathway in prostate cancer cells, including prostate cancer stem cell-like cells, is a promising therapeutic approach and that galiellalactone is an interesting compound for the development of future prostate cancer drugs.

## Introduction

Prostate cancer is the most commonly diagnosed tumor among men and there is a great need of novel therapies against castration resistant prostate cancer [1]. According to the cancer stem cell theory, only a small subset of cancer cells are capable of tumor growth and recurrence [2], [3]. Prostate cancer stem cells appear to be resistant to conventional cancer therapy and may therefore be involved in advanced prostate tumors and cause relapse and metastasis [4], [5]. Prostate tumors comprise of only 0.1% stem cells but failure to eradicate this cell sub-population may cause regeneration of the tumor and drug resistance and in order to prevent recurrence it is important to target all cell types in the tumor [5], [6], [7], [8]. Novel targeted therapies that target prostate cancer stem cell-like cells are highly warranted.

In the search for specific markers of cancer stem cells, aldehyde dehydrogenase (ALDH) has shown promise as such a marker in different cancers including bladder cancer [9], lung cancer [10], head and neck squamous cell carcinoma [11], breast cancer [12] and prostate cancer [13], [14]. A high expression of ALDH in prostate cancer stem cells has been shown to be positively correlated with Gleason score and inversely correlated with patient survival in prostate cancer patients [13]. High ALDH activity has successfully been used to identify tumor initiating prostate cancer cells and metastases [14].

Signal transducer and activator of transcription 3 (STAT3) is an important transcription factor in many cancer types and it has been shown to be involved in drug resistance and to have anti-apoptotic effects in prostate cancer cells. Constitutively active STAT3 contributes to oncogenesis through upregulation of genes coding for anti-apoptotic proteins, cell cycle regulators and angiogenesis stimulators, leading to increased survival and uncontrolled growth of cancer cells [15]. STAT3 expression is suggested to be correlated to malignant potential and metastatic behavior in prostate cancer [16], [17]. Furthermore, gene expression analysis of prostate cancer stem cells has revealed a pro-inflammatory phenotype and that the JAK/STAT3-signaling pathway is active in this cell population [18]. Several studies highlight STAT3 as a valid target for the development of new drugs for prostate cancer and other malignancies [19], [20], [21]. We have shown, both *in vivo* and *in vitro*, that the STAT3 inhibitor galiellalactone possesses growth inhibitory effects on prostate cancer cells expressing active, phosphorylated STAT3 [22]. Galiellalactone is a metabolite produced by the fungus *Galiella rufa* and it has been synthetically produced as previously described [23]. Galiellalactone is a highly potent and selective inhibitor of IL-6 signaling through STAT3, and is believed to inhibit STAT3 signaling by blocking the binding of activated STAT3 to DNA [24].

In this study we aimed to explore the expression of ALDH as a marker for cancer stem cell-like cells in different human prostate cancer cell lines and the effects of the STAT3 inhibitor galiellalactone on ALDH expressing prostate cancer cells.

## Materials and Methods

### Cell culture

The human prostate cancer cell lines DU145, LNCaP (from the American Type culture Collection, [ATCC]) and long-term interleukin-6 (IL-6) stimulated LNCaP cells (LNCaP-IL6 cells) [25] were used. The cells were cultured in RPMI 1640 medium supplemented with 10% FBS and 1% penicillin-streptomycin. LNCaP-IL6 cells were maintained in the above medium supplemented with IL-6 (5 ng/ml; Sigma-Aldrich, St. Louis, MO). All cells were incubated at 37°C in a humidified atmosphere of 95% O_2_ and 5% CO_2_. Cells were used at low passages and not cultured for more than two-three months. Cells were routinely tested for mycoplasma and found free of mycoplasma. Cell identity was not authenticated by the authors. Galiellalactone was prepared by synthesis as previously described [23].

### ALDEFLUOR assay and cell sorting

Prostate cancer cells (DU145, LNCaP and LNCaP-IL6) were subjected to ALDEFLUOR® assay (StemCell Technologies, Aldagen, Inc., Durham, NC) followed by flow cytometry to detect cells with high activity of ALDH (ALDH+). The active reagent BODIPY®-aminoacetaldehyde was added to the cells which was converted by ALDH to the fluorescent BODIPY®-aminoacetate. The ALDH inhibitor diethylaminobenzaldehyde (DEAB) was used as a negative control. DU145 and LNCaP-IL6 cells were treated with 0–50 µM galiellalactone for 24 h and the proportion of ALDH+ was analyzed with ALDEFLUOR® assay. DU145 and LNCaP-IL6 cells were subjected to cell sorting based on the ALDH activity using the ALDEFLUOR® assay. ALDH+ and ALDH− cells were sorted using the BD FACSAria cell sorter (BD Biosciences, San Jose, CA). Non-viable cells were excluded using 7-Amino-actinomycin D staining. ALDH+ and ALDH− subpopulations from DU145 and LNCaP-IL6 cells were re-plated after sorting and analysed with ALDEFLUOR® assay after one week in culture in order to investigate their self-renewing capacity.

### Cytospin and immunohistochemistry

ALDH+ and ALDH− cells sorted from DU145 and LNCaP-IL6 cells were directly subjected to cytospin or replated and treated with galiellalactone, trypsinized and washed in PBS before subjected to cytospin. The cell suspension was placed in a cytospin funnel clamped to a glass slide and spun at 800 rpm for 2 minutes. The slides were air dried, fixed in 4% paraformaldehyde for 10 minutes and permeabilized with 1% Triton-x in Tris buffer, pH 7.6 for 1 h. The slides were subjected to immunohistochemistry using Dako Autostainer Plus and EnVision™+ kit (Dako). The antibodies used were CD44 (BD Pharmingen), integrin α2β1 (Abcam), CD133, (c-PARP), p-STAT3 and p-NF-κB p65 (Cell Signaling Technology).

### Apoptotic cells

Sorted ALDH+ and ALDH− cells from DU145 and LNCaP-IL6 cells were treated with 25 µM galiellalactone for 24 h, subjected to cytospin and stained for the apoptotic marker c-PARP. A minimum of 500 cells were counted from two separate experiments and the number of labeled c-PARP cells were calculated relative the total number of cells. The number of apoptotic cells was expressed as a fraction of the total number of cells.

### WST-1 cell proliferation assay

WST-1 proliferation assay was performed to study the effect of galiellalactone on proliferation and viability of ALDH+ and ALDH− cells sorted from DU145 and LNCaP-IL6 cells. ALDH+ and ALDH− cells were cultured in 96-well plates at the density of 2 000 cells/well in 200 µl of medium. Cells were allowed to set for 24 h. The cells were treated with 0–50 µM galiellalactone for 24 h. Samples were made in triplicate. 20 µl WST-1 solution (Roche Applied Science, Mannheim, Germany) was added per well and incubated at 37°C for 4 h. The absorbance of each well was measured using a scanning multi-well spectrophotometer, ELISA reader at a wavelength of 450 nm and reference wavelength of 690 nm. The results are presented as per cent of untreated control cells.

### Cloning efficiency analysis

ALDH+ and ALDH− cells sorted from DU145 and LNCaP-IL6 cells were cultured in 24-well plates at a density of 200 cells per well. The colony formation was measured after one week. Clones were visualized with Crystal Violet staining and counted. The cloning efficiency was calculated as percent of plated cells.

### Prostate tumor xenograft model

Six to eight-week old male nude NMR1 mice were kept on a 12 h light-dark cycle with access to food and water *ad libitum*. Experimental procedures were approved and carried out according to the guidelines set by the Malmö-Lund Ethical Committee for use and care of laboratory animals. Nude mice with subcutaneous DU145 prostate cancer cell xenografts (1×10^6^ DU145 cells) were subjected to daily i.p. injections of galiellalactone (1 mg/kg) or vehicle for three weeks [22]. After 21 days the mice were sacrificed by ketamin injection (50 mg/kg) followed by cervical dislocation and the xenografts were dissected out and immediately snap frozen in liquid nitrogen and stored at −80°C until further processing. Total RNA was isolated from frozen DU145 xenografts from untreated mice (n = 6) and mice treated with 1 mg/kg galiellalactone per day for three weeks (n = 3).

### Tumorigenicity assay

Freshly sorted ALDH+ and ALDH− populations of DU145 and LNCaP-IL6 cells were suspended in serum free medium/Matrigel (BD Biosciences) mixture (1∶1 volume). Six to eight-week old male nude NMR1 mice (n = 5) were injected with ALDH+ or ALDH− cells subcutaneously in the right and left flank of the mouse, respectively, in concentrations 10 000 or 100 000 cells per injection site. The tumor growth was monitored during the whole experiment. Tumor size was measured after six weeks using a caliper and the tumor volume (µl) was calculated by the formula length (mm)×width×height×0.5632. The mice were sacrificed with isoflurane and cervical dislocation after 6 week.

### Quantitative real-time PCR

The gene expression of CD44, CD133, integrin α2 and ALDH1A1 was investigated with quantitative real-time PCR (q-PCR). Cells were harvested by brief centrifugation directly after cell sorting or after treatment with galiellalactone, and pellets were stored at −80°C until RNA isolation. Total RNA was isolated using QIAshredder spin columns, and further mRNA enrichment and additional washing was performed using the RNeasy kit (Qiagen Sciences). To exclude DNA contamination, samples were DNAse treated for 30 min using RQ1 RNAse-free DNAse (Promega). Complementary DNA (cDNA) was synthesized using random hexamers and reverse transcriptase Superscript II (Invitrogen). Total RNA was isolated from DU145 xenografts using Trizol (Invitrogen). Five micrograms of total RNA was obtained for the cDNA synthesis using a HPLC purified Oligo dTprimer with a T7 sequence.

Quantitative real-time PCR was carried out using 6–20 ng cDNA, 250 nM forward and reverse primer in 2× SYBR Green PCR Master Mix (Applied Biosystems) in a 25 µl reaction. The cycling conditions were: 10 min at 95°C to activate the enzyme, then 40 cycles of 95°C for 15 sec and 60°C for 60 sec. Relative expression levels were quantified by the comparative Ct method [26] and normalized to the expression of the three most stable internal control genes (*HPRT*, *UBC*, and *YWHAZ*), selected by geNorm software analysis as the most stable reference genes. Primers sequences were as previously reported: *HPRT*, *UBC* and *YWHAZ* [26], *ALDH1A1* [27], CD133 [28], CD44 [29], and integrin α2 [30]. All primers were synthesized by Invitrogen and checked for specificity before use.

### Statistics

Results are expressed as the mean ± standard error of the mean (SEM). Statistical analysis of data was performed by Dunnett's test or Student's t test using JMP software (SAS Institute Inc, Cary, NC). The results were considered to be statistically significant at p<0.05.

## Results

### ALDH expression in prostate cancer cell lines

**Figure 1:**
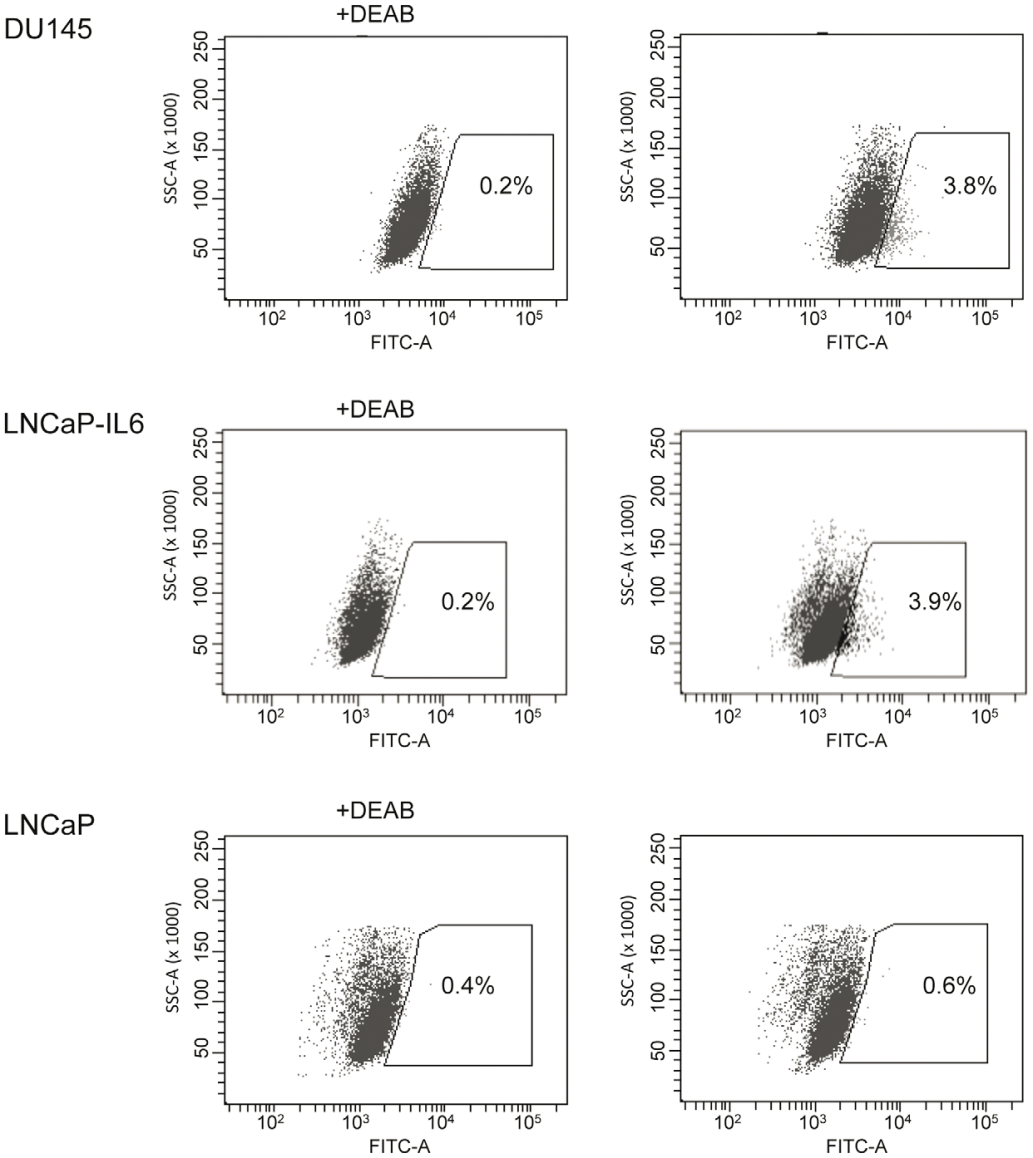
ALDH expressing cells in prostate cancer cell lines. DU145, LNCaP and LNCaP-IL6 cells were subjected to ALDEFLUOR® assay in order to identify cells with high ALDH expression (ALDH+). The ALDH inhibitor DEAB was used as a negative control (left panel). The cells without inhibitor shifted to the right were considered ALDH+ cells (right panel).

DU145, LNCaP and LNCaP-IL6 cells were investigated for their ALDH activity using the ALDEFLUOR® assay and flow cytometry. In DU145 cells 2.62±0.23% of the cells were expressing high ALDH activity (ALDH+), 2.84±0.5% of the LNCaP-IL6 cells were ALDH+ and in LNCaP cells 0.57±0.34% cells showed ALDH activity (n = 3–4) (Figure 1).

### Characterization of isolated ALDH+ cells

The characteristics of ALDH+ and ALDH− cell populations isolated from DU145 and LNCaP-IL6 cells were investigated with respect to self-renewing and colony-forming capacity and tumorigenicity.

The sorted ALDH+ and ALDH− subpopulations from DU145 and LNCaP-IL6 cells were re-plated after sorting and analysed with ALDEFLUOR® assay after one week in culture in order to investigate their self-renewing capacity. After one week in culture small populations of ALDH+ cells were detected among the ALDH− DU145 and ALDH− LNCaP-IL6 cells (0.26±0,24% and 0.36±0.05%, respectively, n = 2). However, after one week in culture after sorting, the ALDH+ LNCaP-IL6 and ALDH+ DU145 cell cultures re-established their parental phenotype and constituted a population resembling that of the original parental cell line with 2.24±1.23% and 1.24±0.14% ALDH+ cells, respectively (n = 2).

**Figure 2:**
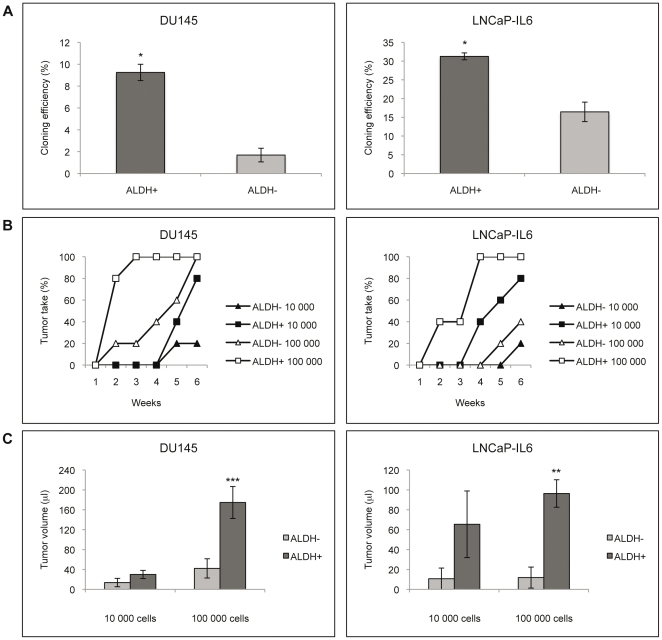
Characteristics of ALDH+ and ALDH− cells. ALDH+ cells show stem cell-like characteristics in terms of increased colony forming capacity and tumorigenicity. A. Clonogenic assay. 200 cells/well were seeded and colonies were counted after 1 week. Clonogenicity was calculated as percent colonies formed in relation to cells seeded (n = 2; p<0.05). B. Tumorigenicity assay. Tumor take after subcutaneous injections of 10 000 or 100 000 cells of freshly sorted ALDH+ and ALDH− cells from DU145 and LNCaP-IL6 (n = 5). C. Tumor size of ALDH+ and ALDH− cell xenografts after 6 weeks (n = 5). ALDH+ cell tumors from 100 000 cells injected subcutaneously were significantly larger than the ALDH− cell tumors for both DU145 cells (p = 0.0002) and LNCaP-IL6 cells (p = 0.0077).

The colony-forming efficiency was significantly greater in ALDH+ cells compared to ALDH− cells from both LNCaP-IL6 and DU145 cells (Figure 2A). The ALDH+ clones formed were visibly larger than the ALDH− clones. The tumorigenicity of ALDH+ and ALDH− cells freshly sorted from DU145 and LNCaP-IL6 cells was investigated by injecting 10 000 or 100 000 cells subcutaneously into the flank of nude mice (n = 5) and tumor growth was monitored weekly. ALDH+ cells showed a greater tumor forming capacity compared to ALDH− cells from both DU145 and LNCaP-IL6 cells and the discovery of palpable tumors was delayed in ALDH− cells (Figure 2B). The ALDH+ tumors were significantly larger than the ALDH− tumors after six weeks (Figure 2C).

### Expression of various biomarkers related to stem cells

**Figure 3:**
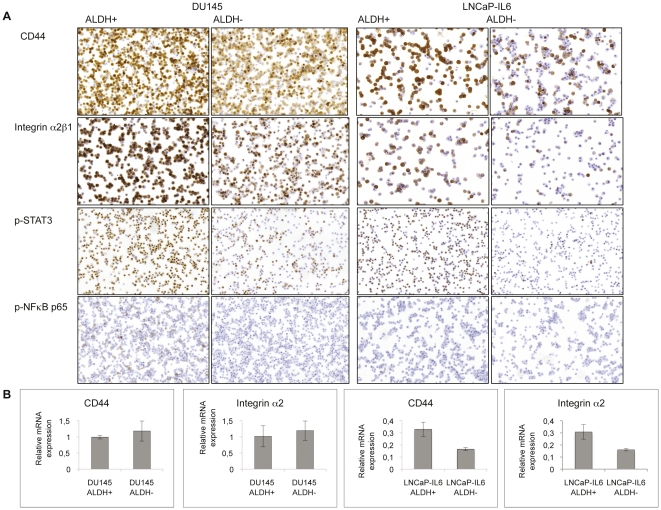
Expression of cancer stem cell related markers. A. Freshly sorted ALDH+ and ALDH− cells from DU145 and LNCaP-IL6 cells were subjected to cytospin and stained for CD44, integrin α2β1, p-STAT3 and p-NF-κB p65. B. The relative mRNA expression of CD44 and integrin α2 in ALDH+ and ALDH− cells freshly sorted from DU1145 and LNCaP-IL6 cells.

The expression of various biomarkers putatively related to cancer stem cells were investigated in freshly sorted ALDH+ and ALDH− fractions of DU145 and LNCaP-IL6 cells (Figure 3). CD44 was detected by immunohistochemistry in both ALDH+ and ALDH− cells sorted from DU145 cells and the expression was more intense in ALDH+ cells compared to ALDH− cells (Figure 3A). CD44 immunostaining was observed in most ALDH+ LNCaP-IL6 cells in contrast to ALDH− LNCaP-IL6 cells where fewer cells were stained for CD44. Integrin α2β1 was expressed in both ALDH+ and ALDH− cells sorted from DU145 cells and the expression was more intense in ALDH+ cells compared to ALDH− cells. Integrin α2β1 was expressed in ALDH+ cells from LNCaP-IL but only a few cells expressed integrin α2β1 in the ALDH− cells. CD133 was not detectable in ALDH+ and ALDH− cells from both DU145 and LNCaP-IL6 cells. The expression of active STAT3 and NF-κB was investigated in ALDH+ and ALDH− fractions of DU145 and LNCaP-IL6 cells (Figure 3A). Increased p-STAT3 expression was observed in ALDH+ cells compared to ALDH− cells from both cell lines. P-NF-κB p65 was detected in ALDH+ DU145 cells but not in ALDH− DU145, ALDH+ LNCaP-IL6 or ALDH− LNCaP-IL6 cells.

The gene expression of CD44, integrin α2 and CD133, was investigated with quantitative -PCR. (Figure 3B). mRNA expression levels of CD44 and integrin α2 was increased in ALDH+ cells compared to ALDH− cells from LNCaP-IL6 cells. There was no difference in the mRNA level of CD44 and integrin α2 in ALDH+ and ALDH− cells from DU145. mRNA levels of CD133 was not detectable in ALDH+ and ALDH− cells from both DU145 and LNCaP-IL6 cells.

### Galiellalactone decreases the proportion of ALDH+ cells

**Figure 4:**
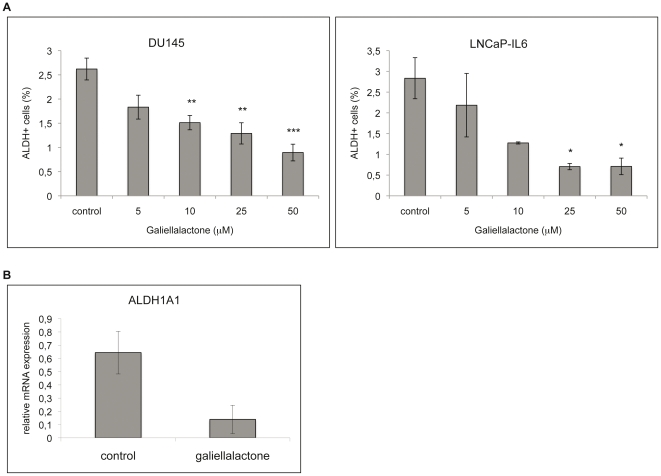
Galiellalactone decreases the proportion of ALDH+ prostate cancer cells in vitro and the ALDH1A1 expression in vivo. A. DU145 and LNCaP-IL6 cells were treated with galiellalactone (5–50 µM) for 24 h and subjected to ALDEFLUOR® assay. The proportion of ALDH+ cells significantly decreased due to galiellalactone treatment in a dose dependent manner. The proportion of ALDH+ cells is expressed as mean ± SEM (n = 2–4). The proportion of ALDH+ DU145 cells were significantly decreased by galiellalactone at the concentrations 10 µM (p = 0.0060), 25 µM (p = 0,0013) and 50 µM galiellalactone (p<0,0001). The proportion of ALDH+ LNCaP-IL6 cells was significantly decreased by galiellalactone at the concentrations 25 µM (p = 0.0441) and 50 µM (p = 0.0445). **B.** Relative mRNA expression of ALDH1A1 in DU145 xenografts in mice treated with 1 mg/kg/day galiellalactone for three weeks. Control mice received vehicle. The relative mRNA expression of ALDH1A1 was reduced in galiellalactone treated mice compared to control (0.139±0.107 and 0.644±0.161, respectively, p = 0.07; n = 3–6).

DU145 and LNCaP-IL6 cells were treated with galiellalactone (5, 10, 25 or 50 µM) for 24 h and subjected to ALDEFLUOR® assay in order to investigate the effect of galiellalactone on the proportion of ALDH+ cells compared to vehicle. Galiellalactone decreased the proportion of ALDH+ cells of both DU145 and LNCaP-IL6 cells in a dose dependent manner (Figure 4A). Incubation with galiellalactone at 25 µM for 24 h significantly decreased the proportion of ALDH+ DU145 cells by 51% (p = 0.0013) and the proportion ALDH+ LNCaP-IL6 cells by 75% (p = 0.0441).

### Galiellalactone decreases the ALDH1A1 mRNA expression in DU145 xenografts

Mice with DU145 xenografts were treated with vehicle or 1 mg/kg galiellalactone daily for three weeks, the tumors were harvested and the gene expression was analyzed. The mRNA expression of *ALDH1A1* was 4.6 times lower in galiellalactone treated xenografts compared to controls (Figure 4B).

### Galiellalactone inhibits proliferation and induces apoptosis of ALDH+ cells

**Figure 5:**
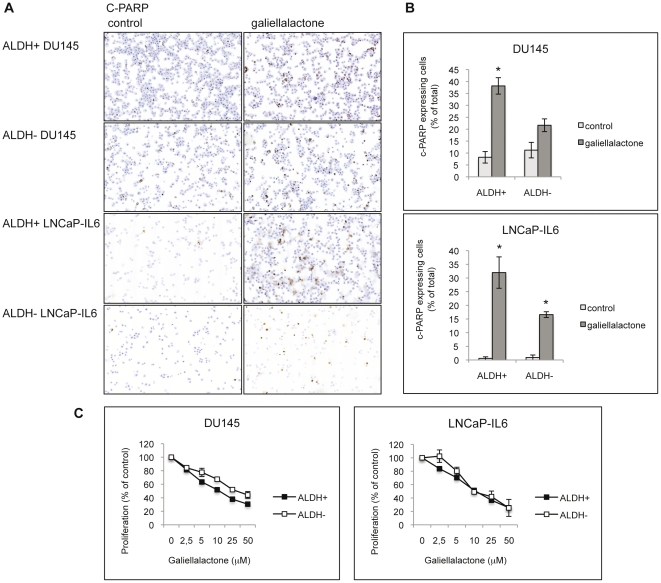
Galiellalactone induces apoptosis and inhibits proliferation of ALDH+ prostate cancer cells. A. ALDH+ and ALDH− cells sorted from DU145 and LNCaP-IL6 cells were treated with 25 µM galiellalactone for 24 h. Apoptotic cells were detected by c-PARP staining. B. Quantification of c-PARP stained apoptotic cells in ALDH+ and ALDH− cells sorted from DU145 and LNCaP-IL6 cells and treated with 25 µM galiellalactone for 24 h. Treatment with galiellalactone significantly increased the amount of c-PARP expressing cells in ALDH+ DU145, ALDH+ LNCaP-IL6 and ALDH− LNCaP-IL6 cells compared to untreated controls (p = 0.019, 0.035 and 0.009 respectively; n = 2). The difference in apoptotic response between galiellalactone treated ALDH+ and ALDH− cells from DU145 and LNCaP-IL6 cells was not significant (p = 0.059 and p = 0.119, respectively; n = 2). C. Galiellalactone decreased the viability of ALDH+ and ALDH− cells sorted from DU145 and LNCaP-IL6 cells. The ALDH+ DU145 cells were significantly more sensitive to galiellalactone compared to the corresponding ALDH− cells at 5 µM (p = 0.0017), 10 µM (p = 0.0010), 25 µM (p = 0.0019) and 50 µM (p = 0.0025). Results are presented as mean per cent of untreated control (n = 2).

Sorted ALDH+ and ALDH− cells from DU145 and LNCaP-IL6 cells were treated with 25 µM galiellalactone for 24 h and immunostained for the apoptotic marker c-PARP (Figure 5A). An increased number of c-PARP positive cells were detected in galiellalactone treated ALDH+ and ALDH− cells compared to untreated cells (Figure 5A). The apoptotic response in ALDH+ and ALDH− cells sorted from DU145 and LNCaP-IL6 cells treated with 25 µM galiellalactone for 24 h was quantified by counting c-PARP expressing cells (Figure 5B). Treatment with galiellalactone significantly increased the amount of c-PARP expressing cells in ALDH+ DU145, ALDH+ LNCaP-IL6 and ALDH− LNCaP-IL6 cells compared to untreated controls. The ALDH+ cells showed a greater apoptotic response to galiellalactone compared to ALDH− cells as measured by c-PARP expression. However, this difference was not significant (p = 0.059 for DU145 cells and p = 0.119 for LNCaP-IL6 cells, n = 2). The effect of galiellalactone on the viability and proliferation of ALDH+ and ALDH− cells was investigated (Figure 5C). Galiellalactone decreased the proliferation of both ALDH+ and ALDH− cells isolated from DU145 and LNCaP-IL6 cells in a dose dependent manner (Figure 5C). The ALDH+ cells were significantly more sensitive to galiellalactone treatment than the ALDH− cells sorted from DU145 cells. However, there was no significant difference in the sensitivity to galiellalactone between ALDH+ and ALDH− LNCaP-IL6 cells.

## Discussion

Prostate cancer stem cells are shown to be androgen independent and lack expression of a functional androgen receptor [6], [18] which renders them immune to androgen deprivation therapy (ADT). In a mouse model of prostate cancer, increased expression of stem cell markers was observed after ADT [31]. Furthermore, cancer stem cells are resistant to current chemotherapy and ALDH expressing cancer stem cells have shown resistance to and enrichment by paclitaxel treatment [32]. The cancer stem cell characteristics such as slow growth, multidrug resistance, enhanced DNA-repair, high expression of anti-apoptotic proteins [33], and in prostate cancer the lack of response to ADT, make these cells very difficult to target with conventional prostate cancer therapy. New therapeutic modalities must therefore be developed, in order to target both the tumor bulk and the cancer stem cells.

In the present study we identified cancer stem cell-like subpopulations in cultured prostate cancer cell lines which responded to treatment with the STAT3 inhibitor galiellalactone. A small fraction (2–4%) of the cell line populations showed high ALDH activity. These ALDH+ cells revealed stem cell-like characteristics such as increased colony forming and self-renewing capacity and high tumorigenicity as well as expression of putative prostate cancer stem cell markers. These cells responded to treatment with galiellalactone.

The capability of reconstituting a heterogenous mass is a definition of cancer stem cells, and we found that isolated ALDH+ prostate cancer cells were capable of self-renewal and re-establishment of the parental cell line and possessed high tumorigenicity *in vivo* and *in vitro*. The ALDH+ population of LNCaP-IL6 cells had a high expression of the putative prostate cancer stem cells markers CD44 and integrin α2β1 [34] compared to the ALDH− population. However, we did not detect CD133 expression in the ALDH+ or ALDH− prostate cancer cells. This is in agreement with the recent study by Pfeiffer and Schalken [35] suggesting that CD133 is not a marker for stem cells in prostate cancer cell lines. Increased JAK/STAT3 and NF-κB activity is expressed in stem cells and tumor initiating stem-like cells in prostate cancer [18], [36] and our findings of active STAT3 and NF-κB in ALDH+ cells is in accordance with this suggestion. Taken together, our results, and others [14], indicate that ALDH expression can be a means of identifying cancer stem cell-like cells in prostate cancer.

The cancer stem cell-like ALDH+ population was greater in long term IL-6 stimulated LNCaP cells compared to LNCaP cells supporting the view that prostate cancer stem cells show a pro-inflammatory phenotype [18], [37]. Gene expression profiling of prostate cancer stem cells show that the STAT3 signaling pathway is overexpressed in these cells [18] and several studies point to STAT3 as a target for therapeutic intervention in tumor stem cells [38], [39]. This is in line with our finding that the ALDH+ stem cell-like cells from prostate cancer cell lines expressed active STAT3 and that these ALDH+ cells responded to the STAT3 inhibitor galiellalactone, which has previously been shown to induce apoptosis of prostate cancer cells with constitutive expression of active STAT3 [22]. The dose-response related decrease of the proportion of ALDH+ cells in the DU145 and LNCaP-IL6 cell populations and induction of apoptosis of these cells upon galiellalactone treatment suggest that these cancer stem cell-like cells are sensitive to STAT3 inhibition. Noteably, DU145 xenografts from galiellalactone treated mice showed reduced gene expression of *ALDH1A1* compared to untreated DU145 xenografts indicating that galiellalactone may target prostate cancer stem cell-like cells also *in vivo*. We have previously showed that the expression of the STAT3 related genes *BCL2L1* and *MCL1* were down-regulated by galiellalactone further confirming the STAT3 inhibitory effect of the drug *in vivo* [22].

Other natural products besides galiellalactone have been shown to inhibit cancer stem cells. Sesquiterpene lactone parthenolide and curcumin are cytotoxic to cancer stem cells and target the cells by inhibiting the activity of NF-κB and STAT3 [40], [41]. Targeting the STAT3 pathway in prostate cancer stem cell-like cells with natural product derived compounds may be a promising therapeutic approach for the development prostate cancer drugs.

In conclusion, prostate cancer cell lines contained ALDH+ subpopulations with stem cell-like characteristics which expressed phosphorylated STAT3. These subpopulations were clearly inhibited by the STAT3 inhibitor galiellalactone. These findings emphasize that targeting the STAT3 pathway in prostate cancer cells, including prostate cancer stem cell-like cells, may be a novel potent treatment strategy in patients with advanced prostate cancer resistant to ADT and cytotoxic therapy and that galiellalactone is an important compound for studying STAT3 signaling in prostate cancer and a potential starting point for the development of future prostate cancer drugs.
